# Emergence of Intrahepatic Cholangiocarcinoma: How High-Throughput Technologies Expedite the Solutions for a Rare Cancer Type

**DOI:** 10.3389/fgene.2018.00309

**Published:** 2018-08-15

**Authors:** Meng-Shin Shiao, Khajeelak Chiablaem, Varodom Charoensawan, Nuttapong Ngamphaiboon, Natini Jinawath

**Affiliations:** ^1^Research Center, Faculty of Medicine Ramathibodi Hospital, Mahidol University, Bangkok, Thailand; ^2^Program in Translational Medicine, Faculty of Medicine Ramathibodi Hospital, Mahidol University, Bangkok, Thailand; ^3^Department of Biochemistry, Faculty of Science, Mahidol University, Bangkok, Thailand; ^4^Integrative Computational BioScience (ICBS) Center, Mahidol University, Nakhon Pathom, Thailand; ^5^Systems Biology of Diseases Research Unit, Faculty of Science, Mahidol University, Bangkok, Thailand; ^6^Medical Oncology Unit, Department of Medicine, Faculty of Medicine, Ramathibodi Hospital, Mahidol University, Bangkok, Thailand

**Keywords:** intrahepatic cholangiocarcinoma, high-throughput technology, integrative multi-omics analysis, molecular biomarker, disease model, translational medicine, precision oncology

## Abstract

Intrahepatic cholangiocarcinoma (ICC) is the cancer of the intrahepatic bile ducts, and together with hepatocellular carcinoma (HCC), constitute the majority of primary liver cancers. ICC is a rare disorder as its overall incidence is < 1/100,000 in the United States and Europe. However, it shows much higher incidence in particular geographical regions, such as northeastern Thailand, where liver fluke infection is the most common risk factor of ICC. Since the early stages of ICC are often asymptomatic, the patients are usually diagnosed at advanced stages with no effective treatments available, leading to the high mortality rate. In addition, unclear genetic mechanisms, heterogeneous nature, and various etiologies complicate the development of new efficient treatments. Recently, a number of studies have employed high-throughput approaches, including next-generation sequencing and mass spectrometry, in order to understand ICC in different biological aspects. In general, the majority of recurrent genetic alterations identified in ICC are enriched in known tumor suppressor genes and oncogenes, such as mutations in *TP53, KRAS, BAP1, ARID1A, IDH1, IDH2*, and novel *FGFR2* fusion genes. Yet, there are no major driver genes with immediate clinical solutions characterized. Interestingly, recent studies utilized multi-omics data to classify ICC into two main subgroups, one with immune response genes as the main driving factor, while another is enriched with driver mutations in the genes associated with epigenetic regulations, such as *IDH1* and *IDH2*. The two subgroups also show different hypermethylation patterns in the promoter regions. Additionally, the immune response induced by host-pathogen interactions, i.e., liver fluke infection, may further stimulate tumor growth through alterations of the tumor microenvironment. For in-depth functional studies, although many ICC cell lines have been globally established, these homogeneous cell lines may not fully explain the highly heterogeneous genetic contents of this disorder. Therefore, the advent of patient-derived xenograft and 3D patient-derived organoids as new disease models together with the understanding of evolution and genetic alterations of tumor cells at the single-cell resolution will likely become the main focus to fill the current translational research gaps of ICC in the future.

## Background

**Figure 1:**
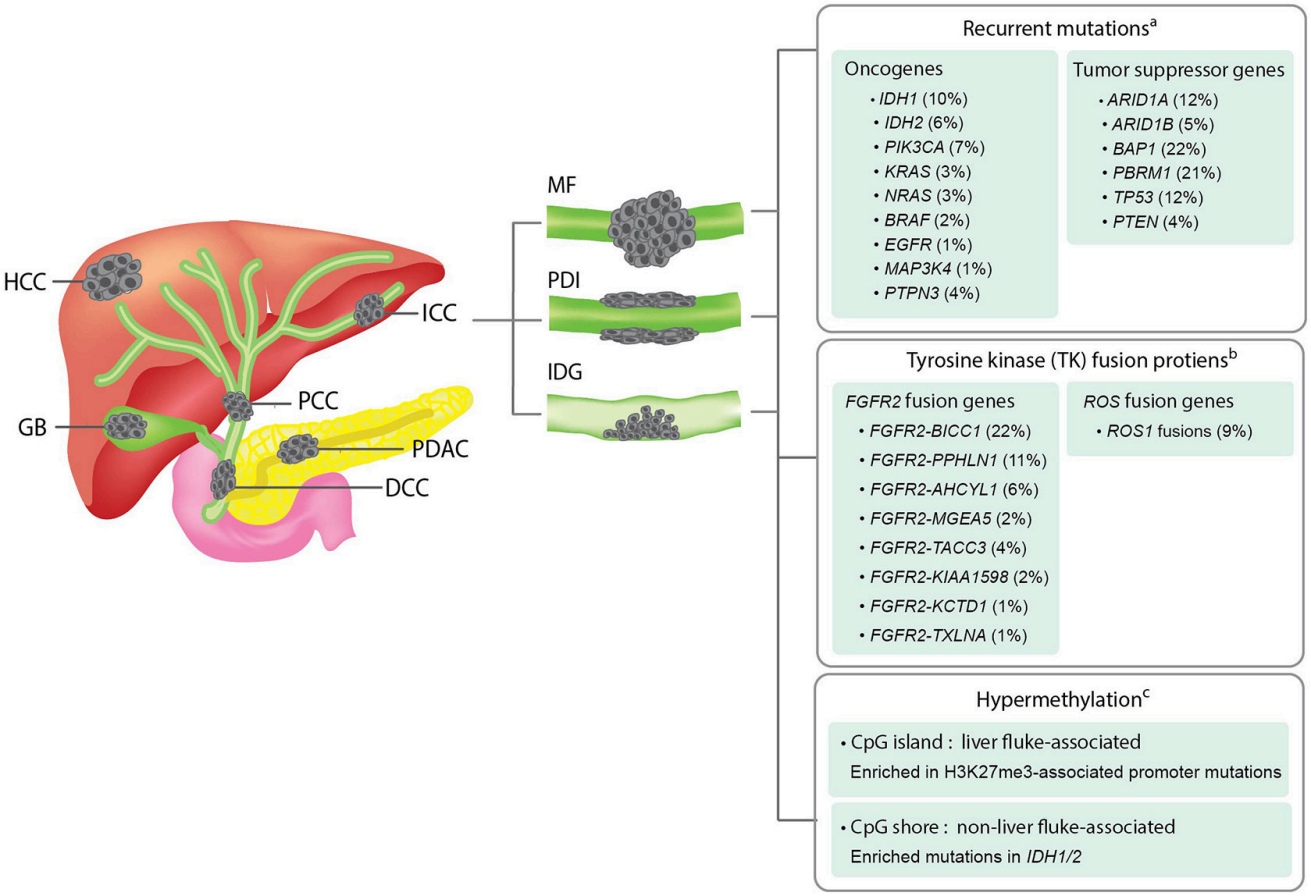
Overview of the anatomical structures, macroscopic subtypes, and recurrent genetic alterations in ICCs. **Left panel;** an illustration showing the anatomical structures of biliary system and their associated malignancies. HCC, hepatocellular carcinoma; ICC, intrahepatic cholangiocarcinoma; PCC, perihilar cholangiocarcinoma; GB, gallbladder cancer; DCC, distal cholangiocarcinoma; PDAC, pancreatic ductal adenocarcinoma. **Middle panel;** an illustration showing the three macroscopic subtypes of ICC. MF, mass-forming type; PDI, periductal-infiltrating type; IDG, intraductal growth type. **Right panel;** a summary of recurrent genetic alterations and their reported frequencies in ICCs. ^a^The mutation frequency of each gene is calculated by dividing the combined number of ICC cases presenting the mutation with the total number of ICC cases analyzed in all four cohorts included in the cBioPortal for Cancer Genomics database (www.cbioportal.org). ^b^The frequency of each fusion gene were obtained from previous literatures (Nakamura et al., 2015; Moeini et al., 2016). ^c^Different hypermethylation patterns of liver fluke-associated and non-liver fluke-associated ICCs and their associated alterations were summarized based on a previous study (Jusakul et al., 2017).

The biliary system includes bile ducts and gallbladder. The main functions of bile ducts are to transfer bile from the liver and gallbladder to the small intestine to help with the digestion and absorption of dietary fats. Bile ducts can be classified into several parts based on the anatomical locations and structures. Peripheral branches of intrahepatic bile ducts drain into the right and left hepatic ducts, which then merge into a larger tube outside the liver, called the common hepatic duct. This extrahepatic bile duct further combines with the cystic duct from the gallbladder and becomes the common bile duct. Cholangiocarcinoma (CCA) is a group of heterogeneous malignancies that occurs in any part of the bile ducts. It can be further classified into three different categories based on the anatomical positions. The tumors that occur in the intrahepatic bile ducts are termed intrahepatic cholangiocar*c*inoma (ICC), while those located between the secondary branches of the right and left hepatic ducts and the common hepatic duct proximal to the cystic duct origin, and in the common bile duct are classified as perihilar and distal cholangiocarcinomas, respectively (Blechacz, 2017; Figure 1). As ICC occurs inside the liver, it is also one of the two main types of primary liver cancers besides hepatocellular carcinoma (HCC). In this review, we aim to provide a comprehensive update and novel insights on ICC, the rare type of CCA, which is known for its extraordinary complexity and heterogeneity, along with dismal prognosis.

Based on a 31-year study in the United States, ICC accounts for only 8% of all CCA cases, and is considered to be a rare disorder (DeOliveira et al., 2007). ICC occurs with the highest prevalence in Hispanic Americans (1.22 per 100,000 people) and lowest in African Americans (0.3 per 100,000 people) (McLean and Patel, 2006). By contrast, it is more common in East Asian and Southeast Asian countries. ICC has an incidence of around 10 per 100,000 people in China (males), and the highest frequency of occurrence, 71 per 100,000 people (males), is found in the northeastern part of Thailand (Shin et al., 2010a). Interestingly, the global incidence of ICC seems to have increased in recent years (Khan et al., 2012).

Risk factors of ICC include bile duct cysts, chronic biliary irritation, parasitic or viral infections, inflammatory bowel disease, abnormal bile ducts, and exposure of chemical carcinogens. Chronic inflammation caused by parasitic infection, particularly liver flukes (*Opisthorchis viverrini* and *Clonorchis sinensis*), is a well-known risk factor of ICC in northeastern Thailand (Sripa et al., 2007; Sripa and Pairojkul, 2008). Eating raw or uncooked fermented fish, a common local dish in this area, results in the high incidence of recurrent liver fluke infections, which are strongly associated with ICC. Several mechanisms have been proposed to explain the association between liver fluke infection and ICC (Sripa et al., 2007). First, when liver flukes start their parasitic life in humans, they attach themselves to the bile duct epithelia using their suckers, which cause damage to the epithelial walls of the ducts. The repeated damage-repair processes may result in the epithelial-mesenchymal transition (EMT) of cell states. Second, the inflammation reactions induced by parasites and the chemicals secreted by them, as well as mutagens from fermented food, may create more carcinogens that damage DNA and result in irreversible oncogenic mutations.

Other than parasitic infections, hepatitis B virus (HBV) and hepatitis C virus (HCV) infections are also associated with ICC. HBV and HCV nucleic acids have been found in 27% of ICC tumors in a US-based study (Perumal et al., 2006). Another study in China has shown a strong association between chronic HBV infection and ICC in a total of 317 patients, and further suggested that ICC and HCC may share a common carcinogenesis process (Zhou et al., 2010). In addition, HBV and HCV infections are proposed to be associated with increasing incidence of ICC from several case-control studies (Yamamoto et al., 2004; Fwu et al., 2011; Sempoux et al., 2011; Yu et al., 2011; Zhou et al., 2012; Li et al., 2015). Other possible risk factors of ICC include smoking, alcohol drinking, obesity and diabetes mellitus, which are mostly observed in western countries (Tyson and El-Serag, 2011). A detailed summary of established risk factors for ICC and their relative risks are shown in Table 1.

**Table 1:** Established risk factors of cholangiocarcinoma.

**Risk factors**	**Relative risk** **(95% CI)**	**References**
**Liver Flukes**		
*Opisthorchis viverrini (*OV*)*^a^	4.8 (2.8–8.4)	Shin et al., 2010b
*Clonorchis sinensis (*CS*)*^b^		
**Viral Hepatitis**		
Hepatitis C virus (HCV)	1.8–4.84	Shin et al., 2010b; Palmer and Patel, 2012
Hepatitis B virus (HBV)	2.6–5.1	
Cirrhosis	5.03–27.2	Tyson and El-Serag, 2011; Palmer and Patel, 2012
Primary Sclerosing Cholangitis (PSC)	Lifetime risk 5–35%	Tyson and El-Serag, 2011
Inflammatory bowel disease (IBD)	1.7–4.67	Tyson and El-Serag, 2011
Obesity	1.56–1.60	Jing et al., 2012; Palmer and Patel, 2012
Type II diabetes	1.43–1.89	Ren et al., 2011; Palmer and Patel, 2012
Hepatolithiasis	5.8–50.0	Tyson and El-Serag, 2011
Congenital abnormalities in biliary tract	10.7–47.1	Tyson and El-Serag, 2011
Alcohol	2.81 (1.52–5.21)	Palmer and Patel, 2012
Genetic polymorphisms^c^	0.23–5.38	Tyson and El-Serag, 2011

a *Endemic in Northeastern Thailand, Lao, Vietnam, Cambodia*.

b *Endemic in South China, Japan, Korea, Taiwan*.

c HFR 677CC+TSER 2R; GSTO1*A140D; MRP2/ABCC2 variant c.3972C>T; (NKG2D rs11053781, rs2617167) +PSC; MICA5.1+PSC; CYP1A2*1A/*1A; NAT2*13,*6B,*7A; XRCCI194W; XRCC1 R280H; PYGS2 Ex10+837 (Tyson and El-Serag, 2011).

The most fundamental categorization of ICC is based on the macroscopic features established by the Liver Cancer Study Group of Japan in 2003 (Yamasaki, 2003). The authors described three macroscopic subtypes of ICC, namely, mass-forming type (MF), periductal-infiltrating type (PDI) and intraductal growth (IDG) type. MF type forms a definite mass in the liver parenchyma. PDI type is defined as tumors that extend longitudinally along the ducts, while the IDG type forms a papillary growth inside the lumen of intrahepatic ducts. MF subtype is the most common subtype (about 65%), whereas PDI and IDG types are less prevalent (around 5% each), and mixed-type (MF+PDI) accounts for ~25% of the cases (Yamasaki, 2003; Sempoux et al., 2011). However, based on more recent data, a noteworthy degree of heterogeneity of ICCs in regard to their histopathological and molecular features was observed. Therefore, in addition to the traditional classifications, multiple new criteria were proposed in order to subcategorize ICC (Vijgen et al., 2017).

Serum biomarkers are usually used to help screen cancer at its earliest stages. A wide variety of markers have been tested in bile and serum with limited success. To date, disease-specific biomarkers for CCA have yet to be established (Valle et al., 2016) and are urgently needed. The most frequently used biomarker for diagnostic and treatment prediction in CCA patients in clinical practice is carbohydrate antigen 19–9 (CA 19-9) (Liang et al., 2015), which is the standard tumor marker for pancreatic adenocarcinoma (Ballehaninna and Chamberlain, 2012). Nevertheless, serum levels of CA 19-9 are also elevated in benign cholestasis such as primary sclerosing cholangitis (PSC), complicating its usage in clinic (Lin et al., 2014). A serum CA 19-9 level >100 U/mL has quite limited sensitivity and specificity (75 and 80%, respectively) in identifying PSC patients with CCA (Chalasani et al., 2000). In ICC, a large cohort analysis by Bergquist et al reported an elevated CA 19-9 level as an independent risk factor for mortality. Elevation of CA 19-9 independently predicted increased mortality with impact similar to node-positivity, positive-margin resection, and non-receipt of chemotherapy (Bergquist et al., 2016).

Since the clinical presentation of ICC is not specific and the disease in its early stage is usually asymptomatic, the patients are often diagnosed at an advanced stage. Surgical resection, which is the only curative treatment, remains the anchor of therapy for patients with resectable ICC (Weber S. M. et al., 2015). Nevertheless, because of the late presentation of symptoms and the central hepatic location of ICC, only ~30% of the patients are deemed eligible for resection by the time of diagnosis. This results in a low 5-year survival and high recurrent rate after resections (Hyder et al., 2013). Loco-regional therapies (LRT) including intra-arterial embolotherapy (IAT) and radiofrequency ablation have been reported as the feasible and effective palliative treatments for patients with unresectable ICC (Savic et al., 2017). Overall, systemic cytotoxic chemotherapy is still the mainstay of treatment for patients with advanced unresectable, recurrent or metastatic ICC. In a landmark phase III randomized study in patients with advanced biliary tract cancer (BTC), doublet chemotherapy (addition of cisplatin to gemcitabine) improved the response rate from 72 to 81% (*P* = 0.049) and overall survival from 8.1 to 11.7 months (hazard ratio 0.64; *P* < 0.001) (Valle et al., 2010). Thus, it has since been considered as the standard of care although the efficacy remains limited. Of note, CCA only accounts for ~60% of all BTC patients enrolled in this study. Another well-established combination chemotherapy regimen for advanced BTC is GEMOX, which consists of gembitabine plus oxaliplatin (Sharma et al., 2010). So far, several clinical trials investigating the efficacy of targeted therapies, such as cetuximab, panitumumab, erlotinib, selumetinib, sunitinib, and bevacizumab, have failed to demonstrate the survival benefits for this group of patients (Zhu et al., 2010; Bekaii-Saab et al., 2011; Jensen et al., 2012; Lee et al., 2012; Yi et al., 2012; Malka et al., 2014).

Taken together, even though ICC is considered a rare cancer type, it represents an emerging health problem with increasing incidence worldwide. ICC is usually diagnosed at late stages and has poor prognosis, partly due to the complex anatomical structure of the biliary system, its various etiologies, heterogeneous subclassifications, and the lack of effective biomarkers and treatments. To date, the genetic signatures of ICC are still limitedly understood and no major driver mutations with clinical actionability have been identified. An overview of current challenges in the treatment of ICC is outlined in Box 1. In the next sections, we aim to provide an in-depth update on the application of recent advances in high-throughput technologies that can help expedite the translation of research discoveries in ICC and related cancers, as well as current disease models used to facilitate the development of precision oncology in ICC.

Box 1 **Challenges in ICC treatment.**

- Intrahepatic cholangiocarcinoma (ICC), a subtype of biliary tract cancer, is considered as a rare disorder with an overall incidence of 1-2 cases per 100,000 people in the US and Europe. However, ICC exhibits vastly different incidence in different parts of the world, mainly based on exposure to the specific risk factors that are common in the regions such as the Southeast Asian liver flukes. The incidence of ICC is currently increasing worldwide.
- Early stages of ICC are usually asymptomatic. The patients are usually diagnosed at advanced stages and metastases are frequently observed. Additionally, a high recurrent rate after tumor resection, which is the sole curative treatment, is also common. The 5-year survival rate for localized disease is only ~15% (American Cancer Society, Inc., 2018).
- The existing serum tumor markers, namely carbohydrate antigen 19–9 (CA19-9) and carcinoembryonic antigen (CEA), lack sensitivity and specificity to detect ICC at an early stage. To date, efficient strategies for the screening and surveillance of ICC have not been established.
- Chemotherapy is a standard of care for advanced disease; however, the efficacy remains limited. Several targeted therapies and their predictive biomarkers have failed to demonstrate survival benefits for this group of patients. Immunotherapy such as checkpoint inhibitor may be effective only in patients with microsatellite instability (MSI), which is uncommon in ICC.
- The highly heterogeneous nature of ICC, comprising both locally advanced and metastatic disease, along with the lack of common genetic alterations and clinically actionable molecular classifications, make it difficult to design the effective clinical trials and assess the efficacy of each treatment regimen. Multiple studies focusing on integrative multi-omics analyses have recently been conducted to identify the molecular classifications of ICC that can help optimize clinical decision.

## Molecular features and subtypes of ICC identified by high-throughput approaches

Advances in high-throughput screening methods such as next-generation sequencing (NGS) and liquid chromatography–mass spectrometry (LC-MS) have enabled broader interrogation of genetic diseases and other disorders. The so-called “omics” data can be defined and categorized according to different groups of biological molecules and regulatory processes, which provide different information of the cells. Given the advantages of broader and deeper scales of available data, different types of omics are applied widely and rapidly to study the associations between different variations and phenotypes, and also used to predict prognosis. It also helps in the classification of subtypes of a disease, which may require different treatment guidelines (Kristensen et al., 2014).

Genomics is one of the earliest to be introduced among the omics data series. Common types of somatic DNA alterations including single nucleotide variants (SNVs), insertions and deletions (INDELs), copy number alterations (CNAs), and structural variations (SVs) have all been shown to play important roles in development and progression of ICC (Zou et al., 2014). Comparative genomics of cancer and normal cells serve as an important platform to investigate molecular mechanisms of cancers; however, biological functions of oncogenes largely depend on how they are expressed (or not expressed) into functional oncoproteins and which tissues they are expressed in. Transcriptomics describes the abundance of transcribed messenger RNA (mRNA) and other non-coding RNAs. Even though most transcriptomic studies on ICC and relating cancers have been focused on mRNA (Jinawath et al., 2006), dysfunction of non-coding RNAs, particularly microRNA (miRNA) and long non-coding (lncRNA), have recently been found to play roles in ICC as well (Wang et al., 2016; Yang et al., 2017; Zheng et al., 2017). Other than transcriptional level, transcriptomic profiling by RNA-Seq data also provides novel information on alternative splicing isoforms of a gene and confirms the expression of novel fusion gene transcripts, which is surprisingly prevalent in ICC (Arai et al., 2014; Borad et al., 2014; Ross et al., 2014; Nakamura et al., 2015; Sia et al., 2015). Transcriptional levels significantly depend on epigenetic configuration of regulatory elements targeting the oncogenes and tumor suppressor genes. It has been shown in CCA, including ICC, that DNA methylation is markedly enriched in either CpG islands or shores, which are regulatory regions enriched in cytosine and guanine nucleotides (Jusakul et al., 2017). Downstream to transcriptomes, proteomics has been widely used to quantify peptide sequences, post-translational modifications, protein abundance and interactions. Aberrant proteins secreted by cancer cells and released into various kinds of body fluids, such as blood, urine and saliva, provide good non-invasive biomarkers for early detection of cancer and the recurrent disease. A few studies have proposed potential biomarkers for CCA and HCC based on mass spectrometry analysis of cancer-specific secreted proteins (Srisomsap et al., 2010; Cao et al., 2013). Another high-throughput approach, metabolomics study, quantifies small molecules, such as amino acids, fatty acids, carbohydrates, or other compounds related to cellular metabolic functions. Metabolite levels and relative ratios reflect metabolic function, and out of normal range perturbations are often indicative of disease, as also shown in ICC (Murakami et al., 2015).

One of the most apparent applications of omic techniques on cancer research is the characterization of cancer subtypes and their signatures, which frequently leads to personalized treatments for cancer patients bearing different tumor signatures. For instance, based on a large whole exome (WES) and genome sequencing (WGS) dataset of 7,042 tumors generated from 30 primary cancer types, cancers could be categorized into 21 different molecular signatures (Alexandrov et al., 2013). Molecular signature 1, for example, has the highest prevalence in all the cancer samples (~70%), and is mostly associated with age. Signature 3 accounts for about 10% of the prevalence and is associated with mutations in *BRCA1*/*2*. Therefore, combining signature 1 and 3 explains over 80% of the breast cancer cases. Even though within each cancer type, the prevalence of somatic mutations varies significantly, they can be distinguished using different combinations of signatures. In parallel, another study categorized 3,299 tumors from The Cancer Genome Atlas (TCGA) comprising 12 cancer types into two main classes, one with dominant oncogenic signatures of somatic mutations (M class), and the others with dominant signatures of CNAs (C class) (Ciriello et al., 2013). The M class tumors show primarily genomic mutations and epigenetic alterations, such as DNA hypermethylation. Conversely, the C class tumors show primarily CNAs, particularly high-level of amplifications and homozygous deletions. Targetable molecular alterations in a tumor class allow the use of class-specific combination cancer therapy. More recently, an integrated analysis of genetic alterations focusing on the 10 canonical signaling pathways in the 9,125 TCGA-profiled tumors from 33 cancer types including CCA has underlined significant representation of individual and co-occurring actionable alterations among these pathways, which suggests targeted and combination therapy opportunities (Sanchez-Vega et al., 2018). In addition, WES and transcriptome data were applied to identify molecular signatures of metastatic solid tumors from 500 adult patients (Robinson et al., 2017). Altogether, such systematic approaches can potentially be applied specifically to ICCs, where each tumor may carry different underlying genetic mechanisms and prognoses, in order to obtain more effective treatment for individual patients.

To overcome the challenges in ICC diagnosis and treatment (Box 1), multiple high-throughput omics studies have been performed in order to discover the underlying molecular mechanisms that can be translated into precision oncology application. In order to better understand the current progress in ICC translational research, here we review the various subclassifications of ICC with regard to its cells of origin, different etiologies and unique clinicomolecular aspects of this rare disorder. The detailed summary of the high-throughput omics studies of ICC can be seen in Table 2.

**Table 2:** Subclassification of ICCs and their associated genetic alterations.

**Classification**	**Technology**	**Subtype I**		**Subtype II**		**References**
		**Class**	**Characters**	**Class**	**Characters**	
Cells of origin	RNA-Seq WES Proteomics	C1 class	- Mutations in *TP53, BAP1, ARID1A, ARID2* - Altered expression of *PLK1* and *ECT2*	C2 class	- Obesity - Bile acid metabolism - T-cell infiltration	Chaisaingmongkol et al., 2017
Anatomical structure	WGS WES RNA-Seq	ICC-specific	- *FGFR2* fusion - *IDH1/2, EPHA2, BAP1* Mutation	ICC and ECC shared	- *KRAS, SMAD4, ARID1A* and *GNAS* mutation	Nakamura et al., 2015
Liver-fluke infection	Microarray	Liver fluke positive	- Xenobiotic metabolism	Liver fluke negative	- Growth factor signaling	Jinawath et al., 2006
	WGS WES WGS		- *TP53, KRAS, SMAD4, MLL3, ROBO2, RNF43, PEG3* and *GNAS* oncogene		- *BAP1, IDH1/*2	Ong et al., 2012; Chan-On et al., 2013
	Epigenomics		- High somatic mutations - *TP53, ARID1A* and *BRCA1/2* mutations - *ERBB2* amplification - Hypermethylation in promoter CpG islands - H3K27me3-associated promoter mutations - Poorer prognosis		- High copy-number alterations - *BAP1* and *IDH1/2* mutations - Altered *PD-1/PD-L2* expression - Alterations and elevated expression of *FGFR* genes - Hypermethylation in promotor CpG shore - Better prognosis	Jusakul et al., 2017
Gene expression and copy number alterations	- Microarray - SNP array	Proliferation class	- Oncogenic pathways - Mutations in *KRAS, BRAF* and *EGFR* - Chr11q13.2 amplification - Chr14q22.1 deletion - Moderate/poorly differentiated - Poorer prognosis	Inflammation class	- Inflammatory pathways (Interleukins/chemokines), - *STAT3* activation signaling pathway - Well differentiated - Better prognosis	Sia et al., 2013a,b Sia et al., 2017
Prognosis	- Microarray	Poor prognosis	- Mutations in *KRAS* and *BRAF*	Good prognosis	- No *KRAS* mutation	Andersen et al., 2013
Mutations and copy number alterations	- WGS - RNA-seq	M class	- Recurrent mutations of *KRAS, TP53, IDH1*	C class	- Recurrent focal copy number alterations including deletions involving *CDKN2A, ROBO1/2, RUNX3* and *SMAD4*	Kim et al., 2016

### Cells of origin of ICC

Primary liver cancer, which is the second leading cause of cancer-related death worldwide, is mainly composed of ICC and HCC. The molecular and clinical features of the two cancers are distinct in most cases. Many studies have shown that the two cancers may share the same driver genes, which may be due to the fact that they also share the same cells of origin; hepatocytes and cholangiocytes arise from a common progenitor, hepatoblasts. ICC usually has poorer prognosis than HCC due to the difficulties in early disease detection and poorly understood carcinogenesis mechanisms. In a small proportion of the cases, ranging from 0.4 to 14% depending on the geographical regions, the patients developed combined hepatocellular cholangiocarcinoma (CHC) (Theise et al., 2010), which was proposed to be of monoclonal origin based on a recent study (Wang et al., 2018).

Various genetically engineered mouse models have been generated to study the cellular origin of primary liver cancers; however, the results are still inconclusive. By ablation of genes in Hippo signaling pathways (Lee et al., 2010; Lu et al., 2010) or knocking out neurofibromatosis type 2 (*Nf2*) gene (Benhamouche et al., 2010) in mouse, the authors proposed that ICC and HCC may share the same progenitor cells since all surviving mice eventually developed both CCA and HCC. A similar result was achieved by performing transduction of oncogenes, i.e., *H-Ras* or *SV40LT*, in mouse primary hepatic progenitor cells, lineage-committed hepatoblasts, and differentiated adult hepatocytes. Regardless of the hepatic lineage hierarchy, transduced cells were able to give rise to a continuous spectrum of liver cancers from HCC to CCA suggesting that any hepatic lineage cell can be cell-of-origin of primary liver cancer (Holczbauer et al., 2013). Several large multi-omics studies have shown that ICC and HCC share recurrently mutated genes including *TP53, BAP1, ARID1A, ARID2* (Chaisaingmongkol et al., 2017; Farshidfar et al., 2017; Wang et al., 2018). Furthermore, ICC together with HCC can be categorized into C1 and C2 subtypes. ICC-C1 and HCC-C1 share similar transcriptomic patterns that are significantly different from those of ICC-C2 and HCC-C2. Interestingly, ICC-C1 and HCC-C1 are enriched for aberrant mitotic checkpoint signaling, suggesting a high rate of chromosomal instability, while C2 groups are enriched for the cell immunity-related pathways, which implies an association with inflammatory responses (Chaisaingmongkol et al., 2017). These findings indicate that ICC and HCC, while clinically treated as separate entities, share common molecular subtypes with similar actionable drivers that can be exploited to improve precision therapy.

It should be noted that ICC- or HCC-specific alterations also exist. Aberrant activation of NOTCH signaling and gain-of-function mutations in the genes encoding isocitrate dehydrogenases (*IDH1* and *IDH2*) are required for ICC development, and thus are significantly more common in ICC than in HCC (Sekiya and Suzuki, 2012; Moeini et al., 2016). In addition, activation of *KRAS* and deletion of *PTEN* in the mouse model will only generate ICC (Ikenoue et al., 2016). Multiple studies have identified different molecular features of ICC and HCC by applying large-scale high-throughput datasets. By combining metabolomics and transcriptomics data from 10 ICC and six HCC samples together with their paired normal tissues, a research team showed that ICC can be distinguished from HCC by the distinct expression patterns of 62 mRNAs, 17 miRNAs, and 14 metabolites (Murakami et al., 2015), leading to the conclusion that ICC and HCC have different oncogenic mechanisms. Recently, Farshidfar et al. conducted a meta-analysis study by combining sequencing data from a total of 458 ICC, 153 pancreatic ductal adenocarcinoma (PDAC), and 196 HCC samples from multiple studies including TCGA. They identified a distinct subtype of ICC enriched for *IDH* mutants, and found that HCC can be characterized by *CTNNB1* and *TERT* promoter mutations, which are absent in ICC (Farshidfar et al., 2017).

In conclusion, although ICC shares some molecular changes with HCC, likely because of the same cells of origin, this rare cancer also possesses its own unique differentiation and evolution pathways, as well as specific genetic alterations and distinct gene expression patterns.

### Different etiologies of ICC

Parasitic infection by liver flukes, i.e., *O. viverrini* (OV) and *C. sinensis*, is a well-known ICC risk factor, particularly in Thailand. The chronic liver fluke infection is estimated to account for 8–10% of the overall ICC incidences (Gupta and Dixon, 2017). The gene expressions studied by Jinawath et al. (2006) was one of the first reports to elucidate the different genetic mechanisms between liver fluke- and non-liver fluke-associated ICCs. Using cDNA microarray, the authors compared the two groups of ICC at the transcriptional level, and found that genes involved in xenobiotic and endobiotic metabolisms, i.e., UDP-glucuronosyltransferase (*UGT2B11, UGT1A10*) and sulfotransferases (*CHST4, SUT1C1*), have higher expression in liver fluke-associated ICCs comparing to non-liver fluke group. These genes are believed to play important roles in detoxification of carcinogens such as nitrosamines from preserved food and, if any, toxic substances released from the parasites or created by parasite-induced chronic inflammation. On the other hand, genes involved in growth factor signaling show higher expression in non-liver fluke ICCs.

Different causative etiologies may induce distinct somatic alterations. Recurrent infection of liver flukes, particularly OV, has been associated with different DNA mutation signatures in ICCs. A WES study demonstrated that the frequently mutated genes in OV-related ICCs comprise both known cancer genes, such as *TP53, KRAS* and *SMAD4*, and newly implicated cancer genes including *MLL*3, *ROBO2, RNF43, PEG3*, and *GNAS*, which are genes involved in histone methylation, genome stability, and G-protein signaling (Ong et al., 2012). Another WES study further showed that *TP53* mutations are more enriched in OV-related ICCs, while mutations in *BAP1, IDH1*, and *IDH2* genes are more common in non-OV-related tumors (Chan-On et al., 2013).

A recent multi-omics study analyzed the combined datasets of WGS, WES, CNAs, transcriptomes and epigenomes, and identified four CCA clusters likely driven by distinct etiologies, with separate genetic, epigenetic, and clinical features (Jusakul et al., 2017). The results showed that liver fluke infection is one of the most important classification factors and is also the factor that leads to poorer prognosis. From this study, clusters 1 and 2, which are liver fluke positive, are enriched for recurrent mutations in *TP53, ARID1A* and *BRCA1/2*, and *ERBB*2 amplifications. In contrast, clusters 3 and 4, which comprise mostly non-liver fluke-associated tumors, are enriched for recurrent mutations in epigenetic-related genes, i.e., *BAP1* and *IDH1*/*2*, as well as *FGFR* rearrangements, and have high PD-1/PD-L2 expression. Additionally, DNA hypermethylation of CpG islands and high levels of mutations in H3K27me3-associated promoters were only observed in clusters 1, while cluster 4 exhibited DNA hypermethylation in CpG shores. These findings suggest different mutational pathways across all four CCA subtypes.

Other than liver fluke, hepatitis virus infection has been proposed to be associated with an increased risk of ICC as well. A meta-analysis of the combined 13 case–control studies and three cohorts of ICC patients has reported a statistically significant increased risk of ICC incidence with HBV and HCV infection (OR = 3.17, 95% CI, 1.88–5.34, and OR = 3.42, 95% CI, 1.96–5.99, respectively) (Zhou et al., 2012). To investigate whether viral hepatitis-associated ICC may harbor specific histomorphological and genetic features, Yu et al. analyzed the 170 ICC patients who were either seropositive or seronegative for HBV or HCV. The authors identified N-cadherin as an immunohistochemistry (IHC) marker for viral hepatitis-associated ICC. N-cadherin IHC positivity is also strongly associated with cholangiolar morphology, lack of CEA, high MUC2 expression, and low *KRAS* mutation frequency (Yu et al., 2011). In line with these findings, another study conducting WES in ICCs found that HBV-associated ICCs carry high *TP53* mutation loads, while mutations in *KRAS* are almost exclusively identified in tumors of HBV-seronegative patients (Zou et al., 2014). However, larger scale high-throughput studies have yet to be performed in viral hepatitis-associated ICCs.

### Other molecular and clinical aspects

Based on gene expression and SNP microarrays, two main subtypes of ICC, proliferation (PF) and inflammation (IF), were identified (Sia et al., 2013a). The PF subtype is more common and can be characterized by activation of oncogenic signaling pathways, DNA amplifications of 11q13.2 (including *CCND1* and *FGF19* gene loci), deletions of 14q22.1 (including *SAV1* gene locus), mutations in *KRAS* and *BRAF*, and is associated with a poor prognosis. In contrast, the IF subtype is characterized by activation of inflammatory signaling pathways, overexpression of cytokines and STAT3 activation, and is associated with a better prognosis. Another study led by Anderson et al. classified ICC patients into two subgroups based on 5-year survival rate, time to recurrence, and the absence or presence of *KRAS* mutations. Similarly, *KRAS* mutations are associated with poor clinical outcomes (Andersen et al., 2013).

As mentioned earlier, based on a large-scale TCGA study, mutational signatures can be divided into two major classes, namely M and C (Ciriello et al., 2013). By combining WES and transcriptomic data, a study showed that ICCs carry signatures of both M and C classes as well (Kim et al., 2016). ICC of C class harbors recurrent focal CNAs including deletions involving *CDKN2A, ROBO1, ROBO2, RUNX3*, and *SMAD4*, while those of M class harbor recurrent mutations in the genes frequently mutated in ICC, i.e., *TP53, KRAS*, and *IDH1*, as well as epigenetic regulators and genes in TGFβ signaling pathway.

Focusing on the genomic findings from all ICC studies discussed above, recurrent mutations of ICC are enriched in tumor suppressor genes, i.e., *ARID1A, ARID1B, BAP1, PBRM1, TP53, STK11*, and *PTEN*, and oncogenes, i.e., *IDH1, IDH2, KRAS, BRAF*, and *PIK3CA*. The frequencies of these recurrent mutations in ICC across multiple studies are summarized in Figure 1. The majority of these genes are associated with genome instability and epigenetic alterations, which are the common underlying mechanisms of cancer. Recurrent mutations of *BRCA2, MLL3, APC, NF1*, and *ELF3* tumor-suppressor genes have also been reported in ICC (Farshidfar et al., 2017). Using transcriptomic analysis, fibroblast growth factor receptor 2 (*FGFR2*) fusion genes, i.e., *FGFR2-AHCYL, FGFR2-BICC1 type1, FGFR2-BICC1 type2, FGFR2-PPHLN1, FGFR2-MGEA5, FGFR2-TACC3, FGFR2-KIAA1598, FGFR2-KCTD1, and FGFR2-TXLNA*, are found to be one of the most prevalent alterations in ICC (Jiao et al., 2013; Borad et al., 2014; Ross et al., 2014; Murakami et al., 2015; Sia et al., 2015; Farshidfar et al., 2017; Figure 1). Furthermore, they are reported to be exclusively present in ICC, but not ECC and gallbladder cancer (Nakamura et al., 2015). FGFR2 fusion proteins have been shown to facilitate oligomerization and FGFR kinase activation, resulting in altered cell differentiation and increased cell proliferation (Wu et al., 2013). Although the genomic and transcriptomic analyses of ICC support the use of targeted therapeutic interventions, there is currently no targeted therapy considered effective for this disorder. In order to develop a strategy to overcome this challenge, a disease model that mimics most or all biological and genetic aspects of ICC is an ideal tool for performing functional studies of the target genes or screening potential anticancer drugs. In the coming sections, we will update the recent progress and introduce new disease models that may expedite the discovery of novel treatment for ICC.

## Current disease models of ICC

The first ICC cell line, HChol-Y1, was established in 1985. The cell line secretes very low levels of CEA and high level of CA 19-9, which are the markers of various kind of cancers (Yamaguchi et al., 1985). Since then, many more ICC cell lines originating from ICCs with different etiologies have been established around the world. PCI:SG231 (Storto et al., 1990), CC-SW-1 (Shimizu et al., 1992), CC-LP-1 (Shimizu et al., 1992) cell lines were established from patients in the US. HuH-28 (Kusaka et al., 1988), KMCH-2 (Yano et al., 1996), RBA (Enjoji et al., 1997), SSP-25 (Enjoji et al., 1997), NCC-CC1, NCC-CC3-1, NCC-CC3-2, and NCC-CC4-1 (Ojima et al., 2010) were derived from Japanese patients. SNU-1079 (Ku et al., 2002) was derived from a Korean patient, while HKGZ-CC (Ma et al., 2007), and HCCC-9810 (Liu et al., 2013) were derived from Chinese patients. In particular, HuCCA-1 was established from the tumor removed from a Thai patient with liver fluke infection (Sirisinha et al., 1991). This cell line is from epithelial cell origin and secretes a number of non-specific tumor markers including CA125 (Srisomsap et al., 2004).

**Figure 2:**
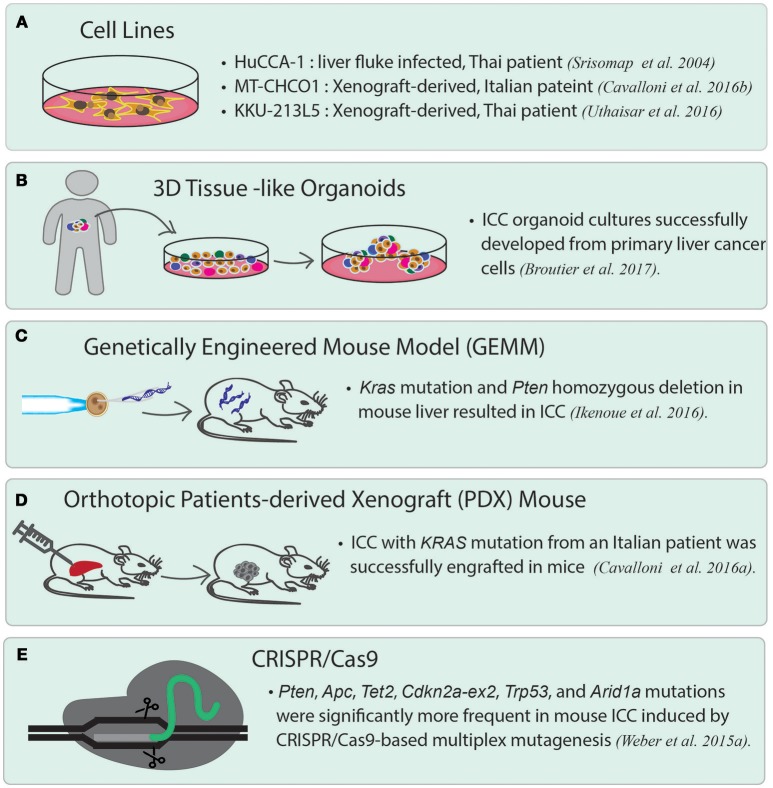
Current disease models for studying ICC. **(A)** ICC cancer cell lines. There are many cell lines established from primary tumor cells. Three representative cell lines are listed. HuCCA-1 was derived from a Thai patient with liver fluke infection. MT-CHCO1 and KKU-213L5 were both established from patient-derived xenografts (PDX). **(B)** 3D patient-derived tissue-like organoids. Organoids preserve the properties of primary tumor cells as well as tissue heterogeneity. **(C)** Genetically engineered mouse model (GEMM). A GEMM of ICC was generated by inducing oncogenic *KRAS* mutation and homozygous *PTEN* deletion in mouse liver. **(D)** Orthotopic patient-derived xenograft (PDX). In orthotopic PDX mouse models, patient-derived tumor cells are transplanted into the same organ from which the patient's cancer originated, followed by stabilizing the tumors in the animals. **(E)** A mouse model of ICC created by CRISPR/*Cas9* gene editing. CRISPR/*Cas9* is used to introduce mutations to the selected tumor suppressor genes including *Arid1a, Trp53, Tet2, Pten, Cdkn2a, Apc, Brca1/2*, and *Smad4*, which lead to ICC in the gene-edited mice.

Unlike most of the ICC cell lines established directly from primary tumor cells, two cell lines, namely MT-CHC01 and KKU-213L5, were established by generating xenograft, which is the growing of human primary tumor cells in the immunodeficient mice, such as nonobese diabetic (NOD)/Shi-severe combined immunodeficient (*scid*)-IL2rγ^*null*^ mice (NOG mice). MT-CHC01 was established from a xenograft derived from the tumor of an Italian patient. After growing primary tumor cells in NOD/Shi-*scid* mice for four generations, the xenograft was stabilized, and the tumors were resected from mice to generate xenograft-derived cell lines. MT-CHC01 retains epithelial cell markers, and shows stemness and pluripotency markers (Cavalloni et al., 2016b). After subcutaneous injection, it retains *in vivo* tumorigenicity and expresses CEA and CA19-9; *KRAS* G12D mutation is also maintained in this cell line. KKU-213L5 was recently derived from its parental cell line, KKU-213, which was established from the primary tumor of a Thai patient. KKU-213L5 was selected *in vivo* through five serial passages of pulmonary metastasized tissues via tail-vein injection into NOD/scid/Jak3 mice (Uthaisar et al., 2016). Compared to KKU-213, KKU-213L5 possesses higher metastatic behaviors, such as higher migration and invasion abilities, and also shows stem cell characteristics. The cells exhibit significantly higher expression of anterior gradient protein-2 (*AGR2*) and suppression of *KiSS*-*1*, which are associated with metastasis in the later stages of disease (Figure 2A).

Recently, the use of human tumor xenograft or patient-derived xenograft (PDX) provides a “patient-like” environment in animal models for a better study of human cancers. To generate PDX, tumor cells are transplanted into immunocompromised animals either by subcutaneous injection or by injecting into the desired organs directly. An orthotopic xenograft model is generated by either implanting or injecting human tumor cells into the equivalent organ from which the cancer originated. It is widely believed that orthotopic PDX reflects the original tumor microenvironment much better than the conventional subcutaneous xenograft models. Recently, a novel PDX model was generated from an Italian patient with ICC. This PDX shows the same biliary epithelial markers, tissue architecture, and genetic aberrations as the primary tumor (Cavalloni et al., 2016a) (Figure 2D). Other than PDX, a genetically engineered mouse model of ICC has been generated by inducing oncogenic *Kras* mutation and homozygous *Pten* deletion in the liver. The tumors induced in this model are exclusively ICCs and show histological phenotype similar to human ICC with cholangiocyte origin. This mouse line is suited for the development of new therapies for ICCs with an oncogenic *KRAS* mutation and the activated PI3K pathway (Ikenoue et al., 2016) (Figure 2C). The latest gene-editing technology, CRISPR/*Cas9* technique, has successfully been used to induce ICC in mice. A study led by Weber J. et al. (2015) introduced mutations in a set of tumor suppressor genes often altered in human ICC/HCC such as *Arid1a, Pten, Smad4, Trp53, Apc, Cdkn2a*, and in a few rarely mutated genes including *Tet2, Brca1/2*, in mice by conducting multiplex CRISPR/*Cas9* gene editing. The results showed that CRISPR/*Cas9*-induced mouse ICCs preferentially carry higher frequencies of mutations in the frequently dysregulated genes in human ICCs, especially those related to chromatin modification. However, the authors unexpectedly observed a high mutation frequency of *Tet2*, which has never been observed in human ICCs. Although *TET2* mutations have not been reported in human ICC, *TET2* is believed to harbor tumor suppressive function linked to *IDH1/2*, which are among the commonly mutated oncogenes in ICC. The authors, therefore, brought up the importance of genetic screening in pinpointing the cancer genes that may not be mutated, but altered by other mechanisms (Weber J. et al., 2015) (Figure 2E).

## Translational clinical aspects and future directions

**Figure 3:**
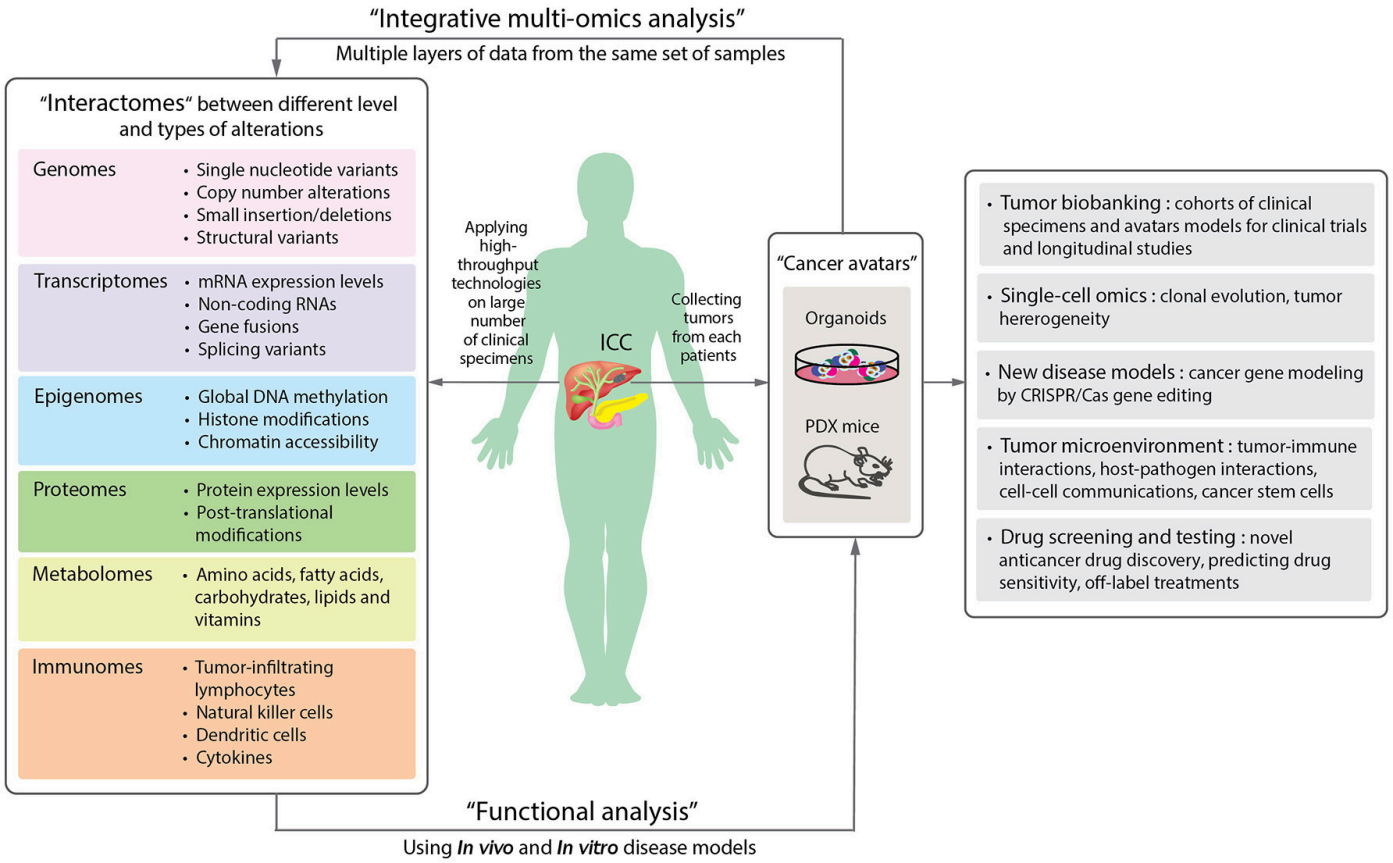
A schematic diagram proposing the application of precision oncology in ICC through the use of high-throughput technologies and disease models. By applying high-throughput technologies on large numbers of patient samples, different levels of omics data can be obtained and provide information of the molecular changes in the tumor cells or microenvironments **(Left panel)**. Aberrant alterations identified from omics data can then be functionally validated in disease models. Organoids and patient-derived xenograft (PDX) mouse are new disease models **(Right panel)**. The two “next-generation” tumor avatars provide “patient-like” models for integrative multi-omics analyses to study the underlying mechanisms of disorders. The avatars can be used for the following studies: single cell sequencing for understanding clonal evolution and heterogeneity of tumors, disease models for gene editing, tumor microenvironments, and high-throughput systematic drug screening and testing. They can further be biobanked for future studies (**Far right panel**).

Looking ahead on the future of cancer research, one of the most exciting trends is the application of patient-derived organoids, which serve as a source of expanded *in vitro* patient-derived cancer cells (Figure 2B). This essentially provides a 3D semi-solid tissue-like architecture that captures the real structure and heterogeneity of a solid tumor, a quality that is lacking in the commonly used immortalized cancer cell lines. Organoid, therefore, serves as a good model for studying the underlying carcinogenesis mechanisms, as well as for drug sensitivity testing and developing targeted therapies (Lancaster and Knoblich, 2014). Recently, human cholangiocytes were isolated and propagated from human extrahepatic biliary tree in the form of organoids as a proof-of-concept experiment for regenerative medicine applications (Sampaziotis et al., 2017). These extrahepatic cholangiocyte organoids can form tissue-like structures with biliary characteristics when transplanted into immunocompromised mice, and can reconstruct the gallbladder wall by repairing the biliary epithelial cells in a mouse model of injury. The results showed that bioengineered artificial ducts can functionally mimic the native common bile duct. Recently, Broutier et al. has successfully developed organoids from primary cell culture of HCC, CHC, ICC, and perihilar CCA (Broutier et al., 2017). By generating ICC organoids that reflect the heterogeneous origins and etiologies, we foresee a possibility of identifying the functions of somatic alterations in ICC by systematically conducting CRISPR/*Cas9* gene editing. In addition, one can investigate the effects of microenvironment more thoroughly (i.e., tumor-immune interactions and cell-cell communications), the cell state transition, and test the efficacy of drugs in a high-throughput manner. Ultimately, patient-derived organoids together with PDX mice may serve as two of the most important models for the development of precision medicine in ICC and other rare cancers. In Figure 3, we summarize the application of precision oncology through the use of high-throughput technologies and disease models to expedite translational research outcomes in ICC.

Intra-tumor heterogeneity reflects the diverse clonal evolution of tumor cells. Tumor evolution is proposed to have one of the following characteristics; hypermutability phenotypes, various mutation signature patterns, weak clonal selection, and high heterogeneity of tumor cell subclones (Schwartz and Schäffer, 2017). Extensive intra-tumor heterogeneity of ICC has lately been observed using WES, which identified branch evolution collectively shaped by parallel evolution and chromosome instability as the predominant pattern of ICC (Dong et al., 2018). As single-cell omics technologies have become more matured recently, it is now possible to characterize the reference expression patterns of individual cells in human (Nawy, 2014) in order to provide the most fundamental knowledge for understanding human health and diseases (Rozenblatt-Rosen et al., 2017). Such advanced technologies will also expedite understanding of carcinogenesis mechanisms, including those of ICC. These approaches include generating transcriptomes and epigenomes at the single-cell level (scRNA-seq and scATAC-seq, respectively), as well as spatial transcriptomes, which can be used to investigate physical relationships of each cell in a tumor mass (Ståhl et al., 2016). Single-cell genomics has also become another important tool for understanding the clonal evolution of tumor cells phylogenetically by exploring the mutating ability of cancer cells (Kim and Simon, 2014; Müller and Diaz, 2017). In the same way, single-cell genomics may help better elucidate the heterogeneity of ICC, particularly when combined with other multi-level omics data generated from either primary tumor cells or the patient-derived 3D tumor model such as organoids. A recent study by Roerink et al. has investigated the nature and extent of intra-tumor diversification at the single cell level by characterizing organoids derived from multiple single cells from three colorectal cancers and adjacent normal intestinal crypts. Interestingly, the responses to anticancer drugs between even closely related cells of the same tumor are markedly different, emphasizing the importance of studying individual cancer cells (Roerink et al., 2018).

With the current advances in NGS technology, the genomic landscapes of ICC have been largely revealed, which is critically important for the clinical development of novel drugs. In addition, the multi-omics profiles that can classify tumor types based on molecular features may be essential for the clinical success in treating the patients. Toward this direction, the clinical trials driven by biomarkers are being conducted. Many ongoing clinical trials of all types of CCA including ICC are listed in Table 3. Among these, targeting FGFR alterations appear to be particularly promising. A phase 2 study of BGJ398, a selective pan-FGFR inhibitor, in metastatic FGFR-altered CCA patients who failed or were intolerant to platinum-based chemotherapy demonstrated impressive anti-tumor activity (Javle et al., 2016). Among the 22 evaluable metastatic patients harboring FGFR2 fusions or other alterations, three patients achieved partial response (PR) and 15 patients had stable disease (SD). A Phase 1 study of ARQ 087, an oral pan-FGFR inhibitor, in patients harboring FGFR2 fusions demonstrated two patients with a confirmed PR and one with durable SD at ≥16 weeks (Papadopoulos et al., 2017). A phase 3 study of ARQ 087 is ongoing and recruiting more patients with FGFR2 fusions as well as inoperable or advanced ICC (NCT03230318). Other novel drugs targeting FGFR fusions such as INCB054828, H3B-6527, erdafitinib, and INCB062079 are in early phases of clinical development (Table 3).

**Table 3:** Ongoing clinical trials of targeted therapy in cholangiocarcinoma^a^.

**Drug**	**Targets**	**Phase**	**Combination**	**Trial number**
**DRIVER MUTATIONS**				
Dasatinib	IDH1/2	II		NCT02428855
AG-120	IDH1	I, III		NCT02073994, NCT02989857
Metformin	IDH1/2	I/II	Chloroquine	NCT02496741
Varlitinib	EGFR (ErbB-1), Her-2/neu (ErbB-2)	II		NCT02609958
Leucovorin and nal-IRI	EGFR, KRAS	II	5-FU	NCT03043547
Niraparib	BAP1	II		NCT03207347
Merestinib	c-Met, HGFR	I	Gemcitabine + Cisplatin	NCT03027284
LOXO-195	NTRK1, NTRK2, NTRK3	I/II		NCT03215511
Trastuzumab Emtansine	HER2	II		NCT02999672
DKN-01	Wnt, DKK1	I	Gemcitabine + Cisplatin	NCT02375880
Copanlisib (BAY 80-6946)	PI3K signaling pathway	II	Gemcitabine + Cisplatin	NCT02631590
Panitumumab	EGF	II	Gemcitabine + Irinotecan	NCT00948935
**FUSION GENE**				
ARQ 087	FGFR2	I/II, II		NCT01752920, NCT03230318
BGJ398	FGFR2	II		NCT02150967
INCB054828	FGFR2	II		NCT02924376
H3B-6527	FGFR4	I		NCT02834780
Erdafitinib	FGFR	II		NCT02699606
Ceritinib (LDK378)	ROS1, ALK	II		NCT02638909, NCT02374489
INCB062079	FGFR4, FGF19	I		NCT03144661
Entrectinib	ROS1, ALK TrkA, TrkB, TrkC	II		NCT02568267
LOXO-101	NTRK fusion	II		NCT02576431
**ANGIOGENESIS**				
Apatinib	VEGFR-2	III		NCT03251443
Ramucirumab	VEGFR-2	II		NCT02520141
Regorafenib	VEGFR, RET, RAF-1, KIT, PDGFRB, FGFR1, TIE2, BRAF(V600E)	II		NCT02053376
Pazopanib	VEGF, PDGFR, FGFR, KIT	II	Gemcitabine	NCT01855724
	VEGFR/PDGFR/Raf MEK MAPK/ERK	I	GSK1120212	NCT01438554
**CHECKPOINT INHIBITOR**				
Durvalumab (MEDI 4736)	PD-L1, PD-1	I	Guadecitabine (SGI-110)	NCT03257761
Pembrolizumab	PD-1	II	Peginterferon alpha-2b (Sylatron)	NCT02982720
	PD-L1, PD-L2 HSP90	I	XL888	NCT03095781
Atezolizumab	PD-L1	II	Cobimetinib	NCT03201458
	PD-L1	I	Gemcitabine+ Cisplatin	NCT03267940
Nivolumab	PD-1, PD-L1 HDAC inhibitor	II	Entinostat	NCT03250273
	CTLA-4 PD-1	II	Ipilimumab	NCT02834013
ABBV-181	PD-1, PD-L1	I	Rovalpituzumab Tesirine	NCT03000257
ABBV-368	OX40	I	Monotherapy or combination with ABBV-181	NCT03071757
**OTHER PATHWAYS**				
RRx-001	G6PD	II	Gemcitabine + Cisplatin	NCT02452970
CX-4945	CK2	I/II	Gemcitabine + Cisplatin	NCT02128282
Melphalan/HDS	Induce covalent guanine N7-N7 intra- and inter-crosslinks and alkylation of adenine N3 of DNA.	II/III	Gemcitabine + Cisplatin	NCT03086993
BBI503	Cancer stem cell (CSC)	II		NCT02232633
Acelarin (NUC-1031)	dFdCDP, dFdCTP	I	Cisplatin	NCT02351765
CX-2009	Tumor-associated antigen (TAA) CD166	I/II		NCT03149549

a *Information acquired from Clinicaltrials.gov*.

Mutations of *IDH1* were reported in up to 25% of CCA (Lowery et al., 2017). AG-120, a highly selective small molecule inhibitor of mutant IDH1 protein, demonstrated a preliminary efficacy in refractory CCA patients with *IDH1* mutations. A phase 1 study of AG-120 reported one patient who achieved PR and five patients with SD >6 months (Lowery et al., 2017). A phase 3 randomized placebo-controlled study of AG-120 in *IDH1* mutation-positive patients is underway (NCT02073994) (Table 3).

Immunotherapy such as checkpoint inhibitor may be effective only in patients with mismatch-repair deficiency (dMMR). In CCA including ICC, incidences of dMMR and/or microsatellite instability-high (MSI-H) were variously reported as quite low (Liengswangwong et al., 2003, 2006; Limpaiboon et al., 2006; Walter et al., 2017). A phase 2 non-randomized study of pembrolizumab, an anti-PD1 antibody, in 41 patients with progressive metastatic carcinoma demonstrated an immune-related objective response rate of 40, 71, and 0% for the patients who have colorectal cancer with dMMR, CCA and other cancers with dMMR, and colorectal cancer with mismatch-repair proficiency (pMMR), respectively (Le et al., 2015). In addition, WES revealed an average of 1,782 somatic mutations for each dMMR tumor compared with only 73 for a pMMR tumor (*P* = 0.007). High somatic mutation loads were also associated with prolonged progression-free survival (PFS) (*P* = 0.02). Hence, dMMR tumors with a large number of somatic mutations may be more susceptible to immune checkpoint blockade, as a result of the substantial amount of new immunogenic antigens produced. Based on these findings, US FDA (Food and Drug Administration) has granted accelerated approval to pembrolizumab in patients with unresectable or metastatic solid tumors with MSI-H or dMMR. A phase 1b study of pembrolizumab (KEYNOTE-028) with 89 advanced biliary tract cancer patients has reported a preliminary efficacy of checkpoint inhibitor (Bang et al., 2015). Overall response rate was observed in ~17% of the patients. Several other ongoing studies of checkpoint inhibitors are being investigated in combination with other drugs including chemotherapy, targeted drugs, and other immunotherapies (Table 3).

## Conclusions

In summary, we have described how the advances in high-throughput technologies have provided a massive amount of information in understanding the genetic mechanisms of disorders, including rare cancers, and in particular, ICC. To be able to effectively utilize such high-throughput methods in cancer research, one should take the following into consideration. First, the determination of clinical information, such as risk factor exposure or etiologies, disease stages, responsiveness to therapy, histology subtypes and anatomical locations, prior to inclusion of the clinical samples is crucial, as it may affect the overall success of downstream analyses. For ICC, liver fluke and hepatitis virus infections are both strongly associated with the disease. Hence, additional information on whether the patients are seropositive for these infections may help better characterize the sample subgroups. Furthermore, ICC can also be subcategorized by macroscopic features, i.e., MF, IDG, and PDI, which rely on accurate pathological determination of the tumor sections. Secondly, insufficient sample size is one of the greatest challenges in studying ICC and other rare cancer types. This cancer in particular is prevalent in certain regions in Asia, such as northeastern Thailand, where most patients are believed to be associated with liver fluke infection. Finding a suitable ICC cohort with adequate sample size is difficult. Earlier studies have combined patients from different countries/geographical regions as well as other different types of BTC e.g., ECC and gallbladder cancer, in order to elucidate the molecular mechanisms and treatment responses. These cohorts, particularly in the form of clinical trials, are consisted of patients and tumors with different genetic backgrounds, which may have resulted in therapeutic failure due to the confounding factors and selection biases. Lastly, the small amount or low quality of source clinical materials limit the comprehensive applications of true “multi-omics” approaches. The majority of previous studies relied on obtaining multiple levels of omics information from different sets of ICC patients. The restricted amount of biological materials from one patient is the main hindrance of performing multiple omics analyses at once to comprehensively investigate the correlation and connections between multiple regulatory processes. Therefore, in addition to a good systematic longitudinal collection of clinical specimens from cancer patients in a tumor biobank, having organoids or PDX mouse models as “cancer avatars” would, at least in part, solve the problem of sample limitation, and should contribute to better omics study design and more effective translational outcomes for rare cancer patients.

## Author contributions

KC, M-SS, and NJ conceived the concept of the review and figures. KC, M-SS, VC, NN, and NJ wrote the manuscript. KC, M-SS, NN, and NJ prepared the tables and figures. All the authors read, reviewed, and approved the final manuscript.

### Conflict of interest statement

The authors declare that the research was conducted in the absence of any commercial or financial relationships that could be construed as a potential conflict of interest.
